# Synthesis of 4-substituted ethers of benzophenone and their antileishmanial activities

**DOI:** 10.1098/rsos.171771

**Published:** 2018-05-16

**Authors:** Arshia, Faiza Ahad, Nida Ghouri, Kanwal, Khalid Mohammed Khan, Shahnaz Perveen, M. Iqbal Choudhary

**Affiliations:** 1 H. E. J. Research Institute of Chemistry, International Center for Chemical and Biological Sciences, University of Karachi, Karachi 75270, Pakistan; 2 Dr. Panjwani Center for Molecular Medicine and Drug Research, International Center for Chemical and Biological Sciences, University of Karachi, Karachi 75270, Pakistan; 3 Department of Clinical Pharmacy, Institute for Research and Medical Consultations (IRMC), Imam Abdulrahman Bin Faisal University, PO Box 1982, Dammam 31441, Saudi Arabia; 4 PCSIR Laboratories Complex, Karachi, Shahrah-e-Dr. Salimuzzaman Siddiqui, Karachi 75280, Pakistan; 5 Department of Biochemistry, Faculty of Sciences, King Abdulaziz University, Jeddah 21412, Saudi Arabia

**Keywords:** 4-hydroxybenzophenone ethers, antileishmanial, *Leishmania major*

## Abstract

Leishmaniasis is a vector-borne protozoan disease; it mainly originates from the bite of sandfly and initiated when parasite is transmitted to human at metacyclic flagellated promastigote form. In the current study, a synthesis of a series of 4-substituted benzophenone ethers **1–20** was carried out in good yields and their *in vitro* antileishmanial activities were also screened. Among synthetic derivatives, 15 compounds **1**, **3**, **5–12**, **15** and **17**–**20** showed antileishmanial activities against promastigotes of *Leishmania major* with IC_50_ values in the range of 1.19–82.30 µg ml^−1^, and the values were compared with those of the standard pentamidine (IC_50 _= 5.09 ± 0.09 µg ml^−1^). Our study identified a series of new antileishmanial molecules as potential leads. Structures of these synthetic compounds were deduced by different spectroscopic techniques, such as ^1^H and ^13^C nuclear magnetic resonance, electron impact and high-resolution electron impact mass spectrometry and IR.

## 1. Introduction

Naturally, benzophenone nucleus is found in the aerial part of *Gentiana verna* L [1] and *Garcinia cochinchinensis* [2]. Benzophenone-containing molecules are extensively used in medicinal and agriculture fields. Numerous pharmacological properties are associated with this nucleus, such as non-nucleoside reverse transcriptase inhibition [3], antineoplastic, cytotoxic [4], anti-inflammatory [5], antibacterial [6], antimicrotuble [7], antifungal [8], and urease inhibitory activities [9], inhibitory effects at low-density lipoproteins [10], tolemerase inhibitor [11], anti-cancer agent [12], signal transducer and activator of transcription protein inhibitor [13]. In addition to various biological activities, benzophenone skeleton is also known to have a wide range of luminescence properties [14,15]. Benzophenone derivatives have significant use in dyes. This nucleus also exhibits good photo-initiator properties [16,17].

Leishmaniasis is among the neglected diseases and according to the surveys of the World Health Organization, 350 million people are suffering from this. Leishmaniasis is also responsible for a high mortality rate worldwide [18,19]. It is a vector-borne protozoan disease, mainly originated from the bite of sandfly. Leishmaniasis is initiated when parasite is transmitted to human at metacyclic-flagellated promastigote form. The main site of action involves reticulo-endothelial system of the host. Based on symptoms, leishmaniasis appears in diffused, cutaneous, mucosal and visceral (Kala Azar) forms [20,21].

Currently, antileishmanial remedies include antimonial drugs, such as tartaremetic (antimony potassium tartrate), urea stibamine, amphotericin B and pentamidines bisamidine [22]. However, adverse side effects of these chemotherapeutic agents have made their use limited [23].

**Figure 1.**
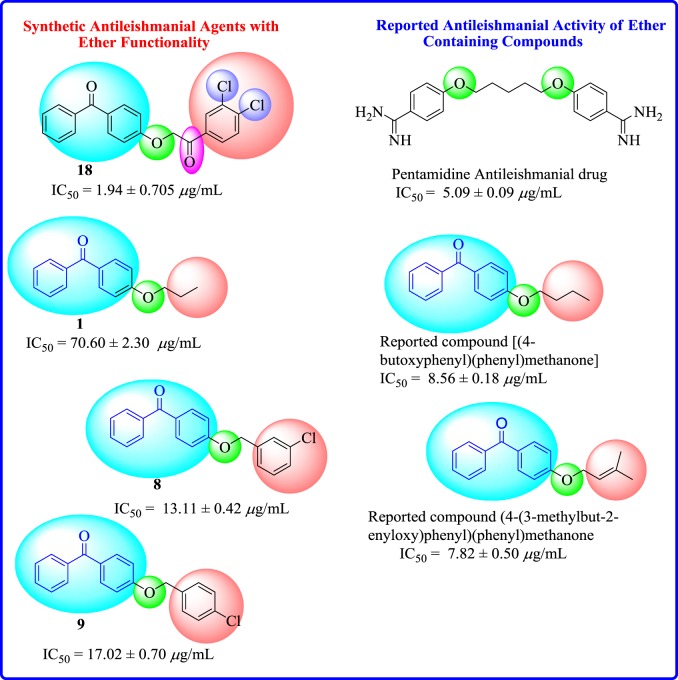
Rationale for the current study.

In the light of a previous report on antileishmanial activities of benzophenone ethers [24], structure of pentamidine which possesses ether functionality (figure 1), and in continuation of our search for antileishmanial agents [25–27], we have synthesized a library of functionalized benzophenone ethers and evaluated their antileishmanial activities *in vitro*. To the best of our knowledge, compounds **1** and **2** were previously reported, while remaining compounds are new [28,29].

## 2. Results and discussion

### 2.1. Chemistry

**Scheme 1.**
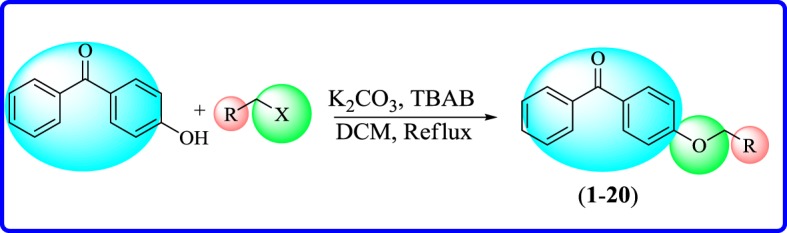
Synthesis of benzophenone ethers.

4-Hydroxybenzophenone (2 mmol), varyingly substituted aryl halide or phenacyl halide (2 mmol), and potassium carbonate (2 mmol) in the presence of catalytic amount of tetrabutylammonium bromide (TBAB) in dichloromethane (15 ml) were refluxed for 6 h. Progression of reaction was studied by thin layer chromatography (TLC). Reaction mixture was cooled to room temperature and a solid material was obtained. The solid was filtered and washed with hexane followed by drying resulting in the desired compounds in good yields (scheme 1). The characterization of synthetic compounds was carried out by ^1^H and ^13^C nuclear magnetic resonance (NMR), electron impact mass spectrometry (EI-MS), high-resolution EI-MS (HREI-MS) and IR spectroscopy.

### 2.2. Spectroscopic studies on representative (most active) compound, 2-(4-benzoylphenoxy)-1-(3,4-dichlorophenyl)ethanone (18)

**Figure 2.**
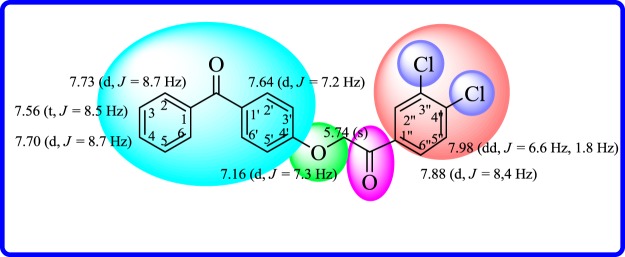
^1^H-NMR chemical shift values for most active compound **18**.

The structure of most active compound (2-(4-benzoylphenoxy)-1-(3,4-dichlorophenyl)ethanone, **18**) was deduced by ^1^H- and ^13^C-NMR spectroscopy which was performed in deuterated dimethylsulfoxide (DMSO-*d*_6_) with a Bruker Avance AM 300 MHz instrument. In ^1^H-NMR spectrum doublet for H-2 and H-6 protons was obtained at *δ*_H_ 7.73 (*J*_2,3/6,5_ = 8.7 Hz). However, one more doublet with integration of two protons at *δ*_H_ 7.64 (*J*_2′,3′/6′,5′_ = 7.2 Hz) was assigned to H-2′ and H-6′. Another proton doublet for H-4 was obtained at *δ*_H_ 7.70 (*J*_4(3,5)_ = 8.7 Hz). A triplet for two protons H-3 and H-5 was obtained at *δ*_H_ 7.56 (*J*_3(2,4)/5(4,6)_ = 8.5 Hz). A doublet at *δ*_H_ 7.88 (*J*_6′′,5′′ _= 8.4 Hz) was assigned to H-6^‴^. However, singlet at *δ*_H_ 5.74 for CH_2_ group confirmed the existence of ether linkage. In addition, other aromatic protons justified their resonance frequency along with their respective *J* values (figure 2).

**Figure 3.**
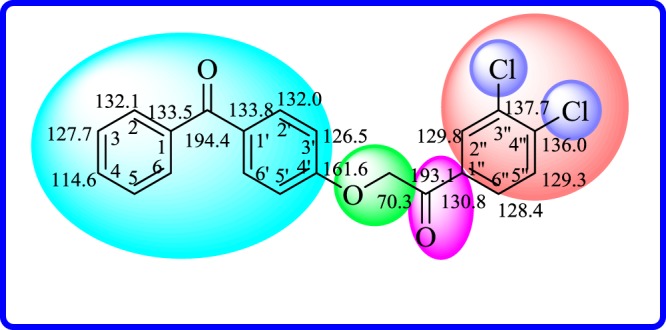
^13^C-NMR chemical shift values for compound **18**.

In broadband decoupled ^13^C-NMR spectra, 16 signals appeared: eight signals are for methines, and seven signals for quaternary carbons. Carbon at *δ*_C_ 70.3 appeared also in spectra: it was for one methylene present in the structure. The most deshielded signals at *δ*_C_ 194.4 and 193.1 were due to carbonylic carbons. Signal at *δ*_C_ 161.6 was due to aromatic cabon directly attached to ether oxygen C-4′. Adjacent to carbonyl groups, three carbons, i.e. (C-1), (C-1′), and (C-1^‴^), resonated at *δ*_C_ 133.5, 133.8 and 130.8, respectively. Rest of the carbons in the structure resonated in the normal aromatic range of *δ*_C_ 132.2–114.6 (figure 3).

**Figure 4.**
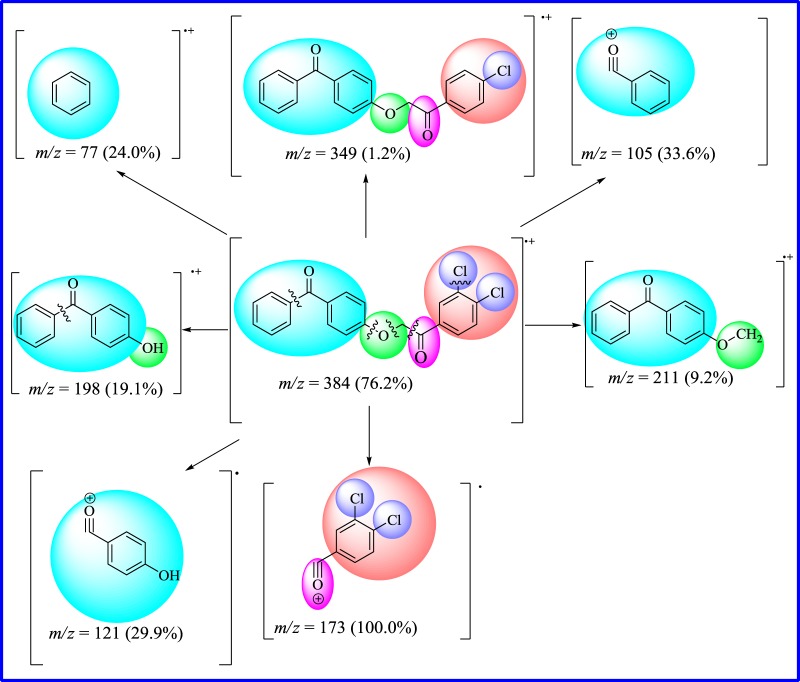
EI-MS fragmentation pattern of compound **18**.

High-resolution mass spectrum of compound **18** displayed the M^+^ at *m/z* 384.0321 with a composition of C_21_H_14_Cl_2_O_3_ (calcd 384.0320). The per cent abundance of isotopic [M + 4]^+^ 10%, [M + 2]^+^ 49% and molecular ion peak M^+^ 76% at *m/z* 388, 386 and 384, respectively, confirmed the presence of two chlorine atoms in a molecule. Cleavage of carbon–carbon bond from *α-*carbonyl group of ether resulted in respective methylene benzophenone ether which appeared at *m/z* 211, and remaining acylium ion appeared as base peak at *m/z* 173. Fragment at *m/z* 198 was due to benzophenone fragment. It was further fragmented into respective acylium ion at *m/z* 121. Fragments at *m/z* 105 and 77 were due to benzyl acylium ion and benzene radical cation, respectively (figure 4).

**Figure 5.**
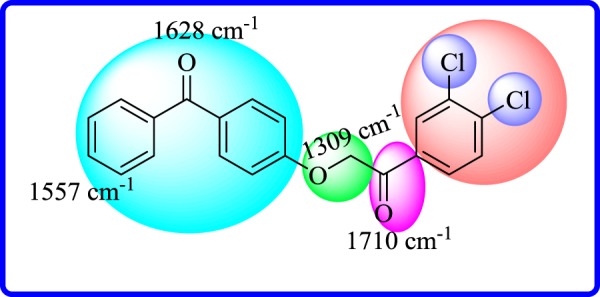
FT-IR absorptions of compound **18**.

In the Fourier transform IR (FT-IR) spectrum, vibrational frequencies at 1710 and 1628 cm^−1^ correspond to the carbonyl (C=O) functionality. However, vibrational frequencies of aromatic (C=C) bond and ether (C–O) appeared at 1557 and 1309 cm^−1^ (figure 5), respectively. These are spectroscopic observations of proposed structure for compound **18**. Structures of all other compounds were deduced in a similar manner.

### 2.3. Antileishmanial studies

**Figure 6.**
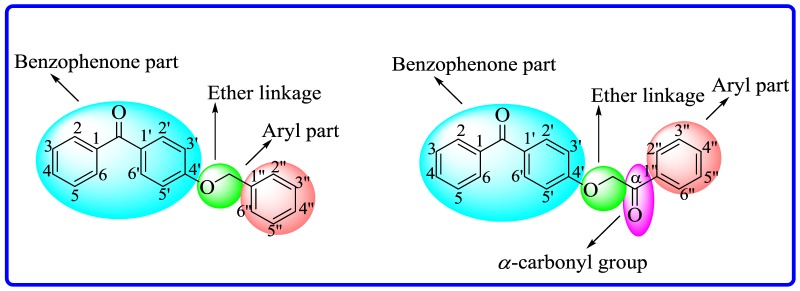
General structures of 4-substituted ether derivatives of benzophenone.

Twenty 4-substituted ether derivatives of benzophenone (**1**–**20**) were synthesized. Among these, nine were α-substituted carbonyl ether derivatives, while 11 were simple ether derivatives of benzophenone. All the synthetic compounds were screened for antileishmanial activities. Results indicated that aryl or alkyl parts of ether analogues having different substituents are responsible for antileishmanial activities (figure 6; table 1).

**Table 1.** Antileishmanicidal activity of benzophenone ethers 1–20 (s.e.m. is the standard error of the mean and n.a. means not active).

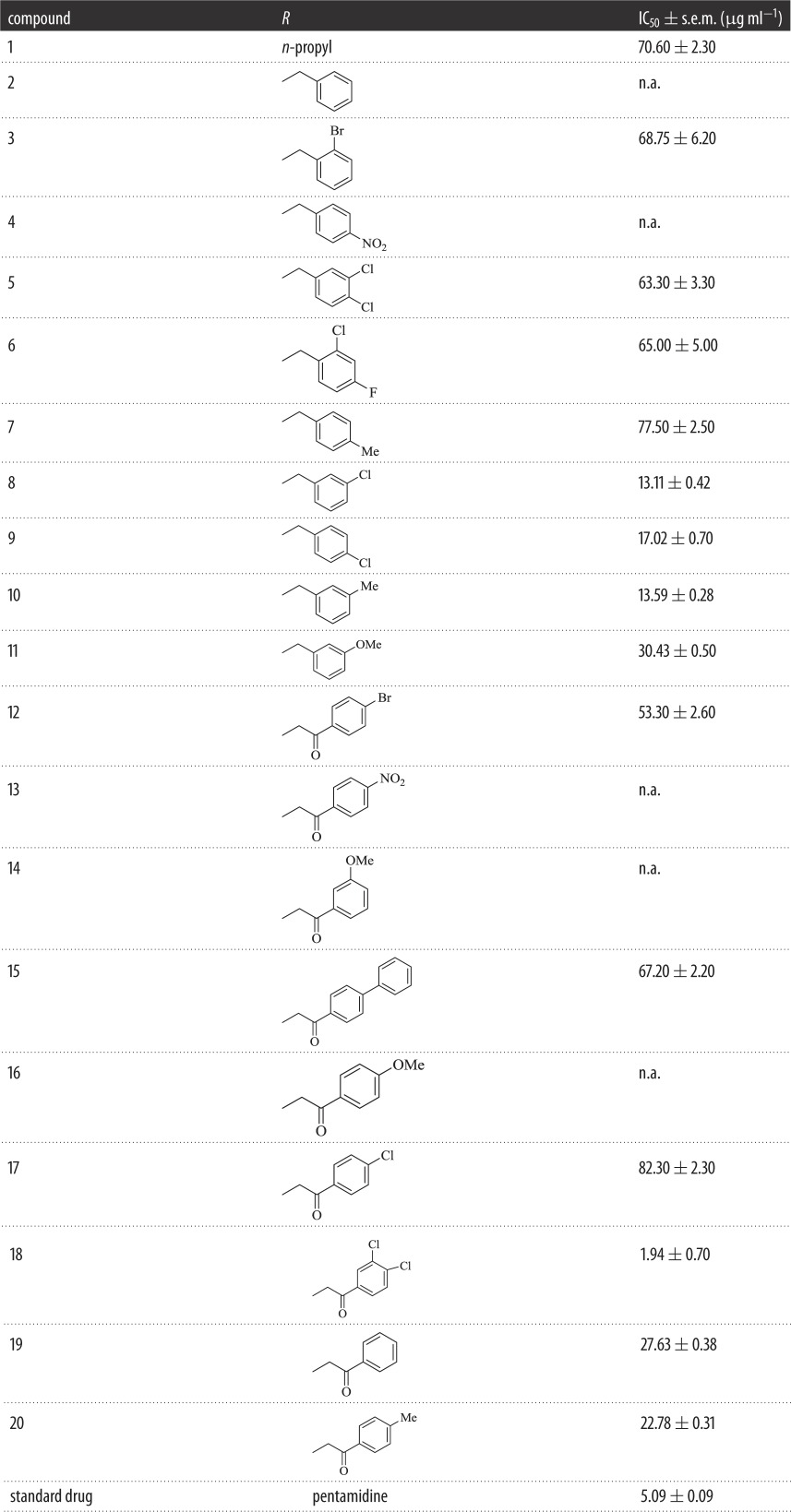

#### 2.3.1. 4-Substituted *α*-carbonyl ether analogues of benzophenone

**Figure 7.**
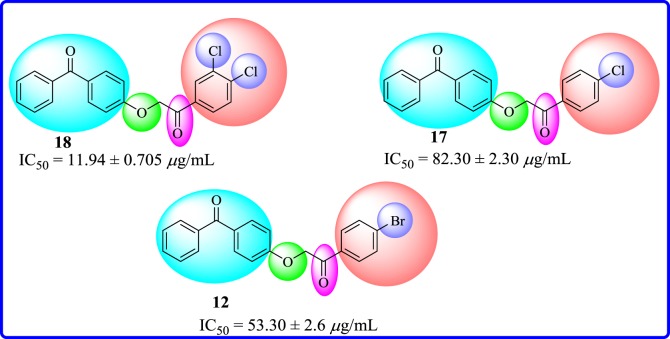
Structure–activity relationship of halide-substituted aryl part for **12**, **17** and **18**.

Among 4-substituted *α*-carbonyl ethers, compound **18** containing chloro groups at *meta* and *para* positions of aryl part was found to be the most active member of series having IC_50_ value of 1.94 ± 0.70 µg ml^−1^. However, the introduction of chloro group at *para* position of aryl part, as in compound **17**, exhibited a decreased inhibitory activity (IC_50_ = 82.3 ± 2.30 µg ml^−1^). Nevertheless, the presence of a bromo functionality at *para* position of aryl part, as in compound **12**, exhibited a weak inhibitory effect (IC_50_ = 53.3 ± 2.6 µg ml^−1^) (figure 7).

**Figure 8.**
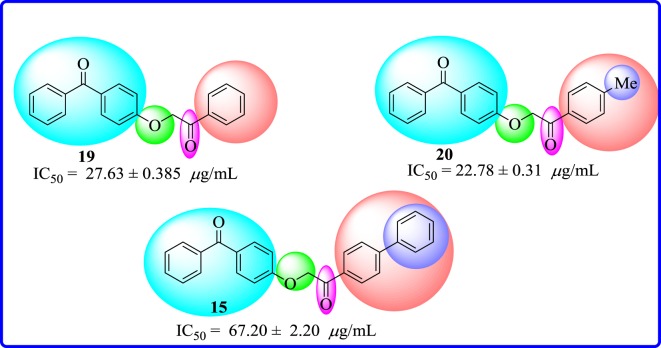
Structure–activity relationship in unsubstituted, methyl and phenyl compounds **15**, **19** and **20**.

When compounds **19**, **20** and **15** were screened for their antileishmanial activities, compound **19** having an unsubstituted aryl part and compound **20** having a methyl group at *para* position of aryl part showed moderate inhibitory effect with IC_50_ value of 27.63 ± 0.38 µg ml^−1^ and 22.78 ± 0.31* *μg ml^−1^, respectively. However, increase of carbon load at aryl part such as placing a *para* phenyl as in analogue **15** resulted in a decreased activity (IC_50_ = 67.2 ± 2.20 µg ml^−1^) (figure 8).

#### 2.3.2. 4-Substituted ether derivatives of benzophenone

**Figure 9.**
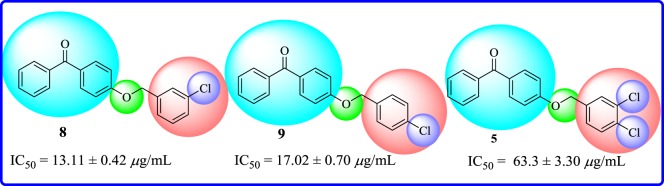
Structure–activity relationship of chloro-substituted compounds **5**, **8** and **9**.

In 4-substituted ether derivatives, compound **8** having chloro group at *meta* position of aryl part was found to be the second most active member of the series with IC_50_ = 13.11 ± 0.42 µg ml^−1^. Nevertheless, the introduction of chloro substituent to *para* position, as in **9** (IC_50_ = 17.02 ± 0.70 µg ml^−1^), showed a slight decreased activity. When a dichloro substituent was present at *meta* and *para* positions of aryl part, as in compound **5**, a sharp decline (IC_50_ = 63.3 ± 3.30 µg ml^−1^) in activity was observed (figure 9).

**Figure 10.**
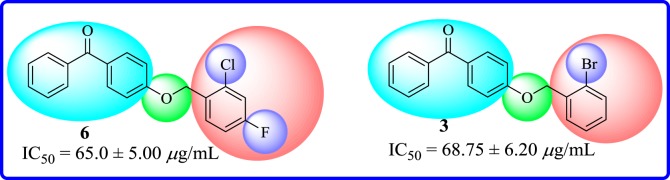
Structure–activity relationship of chloro-, fluoro- and bromo-substituted compounds **3** and **6**.

However, the presence of a chloro group at *ortho* and a fluoro group at *para* as in compound **6** demonstrated a weak inhibitory activity having an IC_50_ value of 65.0 ± 5.00 µg ml^−1^. Moreover, a bromo substituent at *ortho* position, as in compound **3**, made it weakly active with an IC_50_ value of 68.75 ± 6.20 µg ml^−1^ (figure 10).

**Figure 11.**
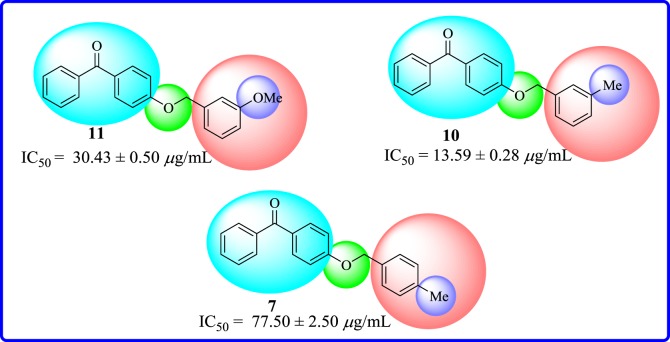
Structure–activity relationship of alkoxy- and alkyl-substituted compounds **7**, **10** and **11**.

The presence of methoxy group at *meta* position as in molecule **11** made it fairly active (IC_50 _= 30.43 ± 0.50 µg ml^−1^). Replacement of methoxy substituent with a methyl substituent at *meta* position of aryl part as in derivative **10** displayed a good activity (IC_50_ = 13.59 ± 0.28 µg ml^−1^). However, when switching the methyl group to *para* position of aryl part as in analogue **7** (IC_50_ = 77.5 ± 2.50 µg ml^−1^), a weak inhibitory activity was observed (figure 11).

**Figure 12.**
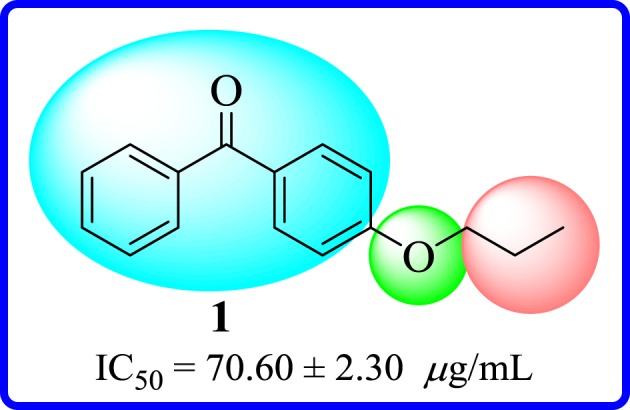
Structure–activity relationship of 4-ether-substituted compound **1**.

To study the effect of carbon load, compound **1** having a propyl group at ether part was screened and found to have a weak activity with an IC_50_ value of 70.6 ± 2.3* *μg ml^−1^. But, remaining derivatives were found to be inactive (figure 12).

## 3. Conclusion

This study deals with the synthesis of 20 4-substituted ethers of benzophenone derivatives and their antileishmanial activities were screened. Fifteen compounds displayed antileishmanial activity having IC_50_ values within the range of 1.94–82.30 μg ml^−1^. Compound **18** was found to be the most active compound (IC_50_ = 1.94 µg ml^−1^) of this series. These compounds seemingly have potential to develop powerful antileishmanial agents.

## 4. Experimental procedure

### 4.1. Material and methods

TBAB, 4-hydroxybenzophenone, potassium carbonate, different phenacyl halide and aryl halides were acquired from TCI (Japan). RPMI 1640 Liquid 20 mM HEPES (4-(2-hydroxyethyl)-1-piperazineethanesulfonic acid), with l-glutamine without NaHCO_3_ was purchased from Sigma-Aldrich (USA). Neubauer counting chamber (2.5 × 10^−3^ mm^2^) was obtained from Marienfeld, Germany. *Leishmania major* was obtained from DESTO. Fetal bovine serum (Cat No. S181H-100 and Lot No. S11302S181H) was acquired from Biowest (The Serum Specialist), and standard drug pentamidine was obtained from Merck. 2-Amino-5-benzonitrile, *N,N*-dimethylformamide 1,1-dimethoxyethane and acetic acid-substituted anilines were purchased from TCI (Japan). All chemicals were used as received without purification. TLC analysis was performed on pre-coated silica gel aluminium cards (Kieselgel 60, 254, E. Merck, Germany). UV lamp at 254 and 365 nm was employed for the visualization of TLC chromatograms.

Mass spectra were recorded with a Finnigan MAT-311A (Germany) mass spectrometer. ^1^H- and ^13^C-NMR spectra were recorded with Bruker Avance AM 300 and 400 MHz spectrometers. Melting points of the compounds were determined using a Stuart^®^ SMP10 melting point apparatus, and are uncorrected. IR spectra (KBr discs) were recorded with a FTS 3000 MX, Bio-RAD Merlin (Excalibur Model) spectrophotometer.

### 4.2. Antileishmanial assay protocol

*Leishmania major* (MHOM/Pk/88/DESTO) was performed in bulk in modified *N,N,N*-biphasic medium by means of normal physiological saline. *Leishmania major* (MHOM/Pk/88/DESTO) promastigotes were grown in the RPMI 1640 medium (Sigma, St Louis, USA), supplemented with 10% heat-inactivated fetal calf serum (PAA Laboratories GmbH, Austria). Parasites at log phase were centrifuged at 2000 r.p.m. for 10 min and at the same speed and washed time three times with saline. Parasites were diluted to a final density of 1 × 10^6^ cells ml^−1^ with a fresh culture medium.

The assay was carried out in a 96-well micro-titre plate; the medium was added in different wells. The test compound (20 µl) was added in the medium and serially diluted. Parasite culture (100 µl) was added in all wells. Two rows were left for positive and negative controls. In positive controls, different quantities of standard antileishmanial drug pentamidine (ICN Biomedical Inc, USA) were present, while negative controls contained only medium. The plate was incubated for 72 h at 22–25°C. The culture was microscopically examined on Neubauer counting chamber. IC_50_ values were calculated by software Ezfit 5.03 (Perella Scientific, USA). All tests were carried out three times [30].

### 4.3. General procedure for the synthesis of compounds 1–20

Differently substituted benzophenone ethers were synthesized by refluxing a mixture of 4-hydroxybenzophenone, potassium carbonate, TBAB, differently substituted phenacyl halide and aryl/alkyl halide in dichloromethane as solvent. The reaction was examined by TLC. Subsequently, the reaction mixture was filtered, and cooled until precipitates became visible. These precipitates were sieved and rinsed with hexane. Yield of all the synthetic compounds was moderate to high.

### 4.4. Spectral data of synthetic compounds 1–20

#### 4.4.1. Phenyl(4-propoxyphenyl)methanone (1)

Yield: 81%; m.p. 91–93°C; R*_f_*: 0.51 (ethyl acetate/hexanes, 2:8); IR (KBr, cm^−1^): 3272 (=C–H), 1645 (C=O), 1599 (C=C), 1257 (C–O); ^1^H-NMR (300 MHz, DMSO-*d_6_*): *δ*_H_ 7.79 (d, 2H, *J*_2,3/6,5_ = 8.7 Hz, H-2, H-6), 7.71 (d, 2H, *J*_2′,3′/6′,5′_ = 6.9 Hz, H-2′, H-6′), 7.63 (t, 1H, *J*_4(3,5)_ = 8.1 Hz, H-4), 7.53 (t, 2H, *J*_3(2,4)/5(6,4)_ = 7.8 Hz, H-3, H-5), 7.03 (d, 2H, *J*_3′,2′/5′,6′_ = 8.7 Hz, H-3′, H-5′), 4.05 (t, 2H, *J*_(CH2,CH2)_ = 6.6 Hz, CH_2_), 1.88 (m, 2H, CH_2_), 1.081 (t, 3H, *J*_(CH3,CH2)_ = 7.5 Hz, CH_3_); EI-MS: *m/z* (rel. abund.%), 240 [M]^+^ (51.0), 198 (20.0), 163 (9.6), 121 (100.0), 105 (14.5), 77 (7.7); HREI-MS: *m/z* calcd for C_16_H_16_O_2_ [M]^+^ 240.1150, found 240.1151.

#### 4.4.2. (4-(Benzyloxy)phenyl)(phenyl)methanone (2)

Yield: 82%; m.p. 90–92°C; R*_f_*: 0.50 (ethyl acetate/hexanes, 2:8); IR (KBr, cm^−1^): 3273 (=C–H), 1641 (C=O), 1597 (C=C), 1242 (C–O); ^1^H-NMR (300 MHz, DMSO-*d_6_*): *δ*_H_ 7.75 (d, 2H, *J*_2,3/6,5_ = 8.7 Hz, H-2, H-6), 7.69 (m, 3H, H-2^′′^, H-4^′′^, H-6^′′^), 7.56 (t, 2H, *J*_3(2,4)/5(4,6)_ = 7.8 Hz, H-3, H-5), 7.48 (d, 2H, *J*_2′,3′/6′,5′_ = 6.9 Hz, H-2′, H-6′), 7.42 (m, 3H, H-4, H-3^′′^, H-5^′′^), 7.18 (d, 2H, *J*_3′,2′/5′,6′_ = 8.7 Hz, H-3′, H-5′), 5.21 (s, 2H, CH_2_); ^13^C-NMR (125.0 MHz, DMSO-*d*_6_): *δ*_C_ 194.3 (C=O), 162.0 (C-4′), 137.7 (C-4), 136.4 (C-1), 132.1 (C-1^′′^), 132.0 (C-1′), 129.5 (C-2′, C-6′), 129.2 (C-2, C-6), 128.5 (C-2^′′^, H-6^′′^), 128.4 (C-3, C-5), 128.0 (C-3^′′^, C-5^′′^), 127.7 (C-4^′′^), 114.6 (C-3′, C-5′), 69.5 (CH_2_); EI-MS: *m/z* (rel. abund.%), 288 [M]^+^ (46.9), 211 (1.4), 198 (4.1), 181 (1.3), 141 (7.0), 121 (8.3), 115 (6.6), 105 (19.7), 91 (100.0), 77 (28.2); HREI-MS: *m/z* calcd for C_20_H_16_O_2_ [M]^+^ 288.1150, found 288.1140.

#### 4.4.3. (4-(2-Bromobenzyloxy)phenyl)(phenyl)methanone (3)

Yield: 84%; m.p. 96–98°C; R*_f_*: 0.47 (ethyl acetate/hexanes, 2:8); IR (KBr, cm^−1^): 3261 (=C–H), 1703 (C=O), 1637 (C=C), 1265 (C–O), 704 (=C–Br); ^1^H-NMR (300 MHz, DMSO-*d_6_*): *δ*_H_ 7.77 (d, 2H, *J*_2,3/6,5_ = 8.7 Hz, H-2, H-6), 7.70 (d, 2H, *J*_2′,3′/6′,5′/3′′,4′′_ = 8.1 Hz, H-2′, H-6′, H-3^′′^), 7.65 (t, 2H, *J*_3(2,4)/5(4,6)_ = 6.9 Hz, H-3, H-5), 7.56 (m, 2H, H-4, H-6^′′^), 7.47 (t, 1H, *J*_5′′ (4′′,6′′)_ = 7.2 Hz, H-5′′), 7.36 (t, 1H, *J*_4′′ (3′′,5′′)_ = 7.5 Hz, H-4_′′_), 7.20 (d, 2H, *J*_3′,2′/5′,6′_ = 8.7 Hz, H-3′, H-5′), 5.23 (s, 2H, CH_2_); ^13^C-NMR (125.0 MHz, DMSO-*d*_6_): *δ*_C_ 194.4 (C=O), 161.8 (C-4′), 137.6 (C-4), 135.2 (C-1), 132.7 (C-1′′), 132.2 (C-1′), 132.1 (C-2′, C-6′), 130.5 (C-2, C-6), 130.4 (C-3, C-5), 129.8 (C-3′, C-5′), 129.3 (C-2′′), 128.4 (C-6′′), 128.0 (C-3′′), 123.0 (C-5′′), 114.6 (C-4^′′^), 69.4 (CH_2_); EI-MS: *m/z* (rel. abund.%), 368 [M + 2]^+^ (8.7), 366 [M]^+^ (8.7), 287 (1.7), 197 (1.0), 181 (0.2), 167 (95.0), 169 (100.0), 152 (1.3), 105 (9.4), 90 (21.7), 77 (11.9); HREI-MS: *m/z* calcd for C_20_H_15_BrO_2_ [M]^+^ 366.0255, found 366.0252.

#### 4.4.4. (4-(4-Nitrobenzyloxy)phenyl)(phenyl)methanone (4)

Yield: 85%; m.p. 135–137°C; R*_f_*: 0.46 (ethyl acetate/hexanes, 2 : 8); IR (KBr, cm^–1^): 3072 (=C–H), 1641 (C=O), 1602 (C=C), 1515 (N=O), 1255 (C–O); ^1^H-NMR (300 MHz, DMSO-*d_6_*): *δ*_H_ 8.28 (d, 2H, *J*_3′′,2′′/5′′,6′′_ = 8.4 Hz, H-3′′, H-5′′), 7.77 (m, 4H, H-2′, H-3′, H-5′, H-6′), 7.69 (m, 3H, H-2, H-4, H-6), 7.56 (t, 2H, *J*_3(2,4)/5(4,6)_ = 7.5 Hz, H-3, H-5), 7.20 (d, 2H, *J*_3′,2′/5′,6′_ = 8.7 Hz, H-3′, H-5′), 5.40 (s, 2H, CH_2_); ^13^C-NMR (125.0 MHz, DMSO-*d*_6_): *δ*_C_ 194.4 (C=O), 161.5 (C-4′), 147.1 (C-4′′), 144.3 (C-4), 137.6 (C-1′′), 132.2 (C-1′), 129.8 (C-1), 129.2 (C-2′, C-6′), 128.4 (C-2, C-6), 128.3 (C-3, C-5), 123.4 (C-2′′, C-6′′), 123.6 (C-3′, C-5′), 114.7 (C-3′′, C-5′′), 69.4 (CH_2_); EI-MS: *m/z* (rel. abund.%), 333 [M]^+^ (43.2), 287 (1.0), 256 (4.1), 197 (30.5), 169 (10.6), 136 (89.7), 106 (74.4), 77 (100.0); HREI-MS: *m/z* calcd for C_20_H_15_NO_4_ [M]^+^ 333.1001, found 333.1014.

#### 4.4.5. (4-(3,4-Dichlorobenzyloxy)phenyl)(phenyl)methanone (5)

Yield: 83%; m.p. 142–145°C; R*_f_*: 0.47 (ethyl acetate/hexanes, 2:8); IR (KBr, cm^−1^): 3255 (=C–H), 1641 (C=O), 1600 (C=C), 1257 (C–O), 810 (=C–Cl); ^1^H-NMR (300 MHz, DMSO-*d_6_*): *δ*_H_ 8.28 (d, 3H, *J*_2,3/6,5/6′′,5′′_ = 8.4 Hz, H-3′′, H-5′′), 7.69 (t, 2H, *J*_3(2,4)/4(3,5)/5(4,6)_ = 7.5 Hz, H-3, H-4, H-5), 7.56 (m, 3H, H-2′, H-6′, H-2′′), 7.18 (d, 2H, *J*_3′,2′/5′,6′_ = 8.7 Hz, H-3′, H-5′), 5.23 (s, 2H, CH_2_); ^13^C-NMR (75.0 MHz, DMSO-*d*_6_): *δ*_C_ 194.3 (C=O), 161.6 (C-4′), 137.7 (C-4′′), 137.6 (C-1′′), 132.1 (C-1′), 131.1 (C-1), 130.7 (C-3′′), 130.5 (C-4), 129.8 (C-2, C-6), 129.6 (C-2′, C-6′), 129.2 (C-2′′), 128.4 (C-5′′), 127.9 (C-6′′), 127.5 (C-3, C-5), 114.7 (C-3′, C-5′), 67.9 (CH_2_); EI-MS: *m/z* (rel. abund.%), 356 [M]^+^ (2.3), 198 (0.2), 159 (100.0), 141 (6.5), 123 (15.3), 105 (11.5), 91 (1.6), 77 (39.7); HREI-MS: *m/z* calcd for C_20_H_14_Cl_2_O_2_ [M]^+^ 356.0371, found 356.0370.

#### 4.4.6. (4-(2-Chloro-4-fluorobenzyloxy)phenyl)(phenyl)methanone (6)

Yield: 82%; m.p. 88–90°C; R*_f_*: 0.45 (ethyl acetate/hexanes, 2:8); IR (KBr, cm^−1^): 3255 (=C–H), 1641 (C=O), 1600 (C=C), 1257 (C–O), 810 (=C–Cl); ^1^H-NMR (300 MHz, DMSO-*d_6_*): *δ*_H_ 8.28 (d, 2H, *J*_2,3/6,5_ = 8.4 Hz, H-2, H-6), 8.26 (s, 1H, H-3′′), 7.69 (t, 3H, *J*_3(2,4)/4(3,5)/5(4,6)_ = 7.5 Hz, H-3, H-4, H-5), 7.56 (m, 4H, H-2′, H-6′, H-5′′, H-6′′), 7.18 (d, 2H, *J*_3′,2′/5′,6′_ = 8.7 Hz, H-3′, H-5′), 5.23 (s, 2H, CH_2_); ^13^C-NMR (75.0 MHz, DMSO-*d*_6_): *δ*_C_ 194.3 (C=O), 161.6 (C-4′), 137.7 (C-4′′), 137.6 (C-1′′), 132.1 (C-1′), 131.1 (C-1), 130.7 (C-3′′), 130.5 (C-4), 129.8 (C-2, C-6), 129.6 (C-2′, C-6′), 129.2 (C-2′′), 128.4 (C-5′′), 127.9 (C-6′′), 127.5 (C-3, C-5), 114.7 (C-3′, C-5′), 67.9 (CH_2_); EI-MS: *m/z* (rel. abund.%), 342 [M + 2]^+^ (2.0) 340 [M]^+^ (5.4), 145 (53.6), 143 (100.0), 141 (6.5), 108 (9.0), 107 (20.8), 105 (9.8) 91 (1.6), 77 (28.4); HREI-MS: *m/z* calcd for C_20_H_14_ClFO_2_ [M]^+^ 340.0666, found 340.0667.

#### 4.4.7. (4-(4-Methylbenzyloxy)phenyl)(phenyl)methanone (7)

Yield: 85%; m.p. 102–104°C; R*_f_*: 0.48 (ethyl acetate/hexanes, 2:8); IR (KBr, cm^−1^): 3091 (=C–H), 1654 (C=O), 1593 (C=C), 1147 (C–O); ^1^H-NMR (300 MHz, DMSO-*d_6_*): *δ*_H_7.74 (d, 2H, *J*_2,3/6,5_ = 8.7 Hz, H-2, H-6), 7.68 (d, 2H, *J*_2′,3′/6′,5′_ = 7.2 Hz, H-2′, H-6′), 7.64 (d, 1H, *J*_4(3,5)_ = 6.3 Hz, H-4), 7.56 (t, 2H, *J*_3(2,4)/5(4,6)_ = 7.5 Hz, H-3, H-5), 7.36 (d, 2H, *J*_3′,2′/5′,6′_ = 8.1 Hz, H-3′, H-5′), 7.21 (d, 2H, *J*_3′′,2′′/5′′,6′′_ = 7.8 Hz, H-3′′, H-5′′), 7.16 (d, 2H, *J*_2′′,3′′/6′′,5′′_ = 8.7 Hz, H-2′′, H-6′′), 5.16 (s, 2H, CH_2_), 2.30 (s, 3H, 4′′-CH_3_); ^13^C-NMR (75.0 MHz, DMSO-*d*_6_): *δ*_C_ 194.5 (C=O), 162.0 (C-4′), 137.7 (C-4′′), 137.3 (C-1′′), 133.3 (C-1′), 132.1 (C-1), 132.0 (C-4), 129.4 (C-2, C-6), 129.2 (C-2′, C-6′), 129.0 (C-2′′, C-6′′), 128.4 (C-3, C-5), 127.9 (C-3′′, C-5′′), 114.6 (C-3′, C-5′), 69.4 (CH_2_), 20.7 (4′′-CH_3_); EI-MS: *m/z* (rel. abund.%), 302 [M]^+^ (11.5), 209 (35.1), 198 (22.5), 179 (5.8), 141 (6.1), 121 (36.6), 105 (100.0), 91 (7.7), 77 (16.8); HREI-MS: *m/z* calcd for C_21_H_18_O_2_ [M]^+^ 302.1307, found 302.1305.

#### 4.4.8. (4-(3-Chlorobenzyloxy)phenyl)(phenyl)methanone (8)

Yield: 81%; m.p. 128–130°C; R*_f_*: 0.46 (ethyl acetate/hexanes, 2:8); IR (KBr, cm^−1^): 3100 (=C–H), 1660 (C=O), 1598 (C=C), 1197 (C–O); ^1^H-NMR (400 MHz, DMSO-*d_6_*): *δ*_H_7.76 (d, 2H, *J*_2,3/6,5_ = 10.0 Hz, H-2, H-6), 7.69 (m, 3H, H-3, H-4, H-5), 7.56 (m, 3H, H-2′, H-6′, H-2′′), 7.45 (m, 3H, H-4′′, H-5′′, H-6′′), 7.18 (d, 2H, *J*_3′,2′/5′,6′_ = 11.6 Hz, H-3′, H-5′), 5.23 (s, 2H, CH_2_); EI-MS: *m/z* (rel. abund. %), 322 [M]^+^ (11.8), 169 (10.6), 141 (18.2), 125 (100.0), 89 (23.9), 77 (25.9); HREI-MS: *m/z* calcd for C_20_H_15_ClO_2_ [M]^+^ 322.0761, found 322.0762.

#### 4.4.9. (4-(4-Chlorobenzyloxy)phenyl)(phenyl)methanone (9)

Yield: 81%; m.p. 130–132°C; R*_f_*: 0.47 (ethyl acetate/hexanes, 2:8); IR (KBr, cm^−1^): 3125 (=C–H), 1670 (C=O), 1634 (C=C), 1278 (C–O), 878 (=C–Cl); ^1^H-NMR (400 MHz, DMSO-*d_6_*): *δ*_H_ 7.75 (d, 2H, *J*_2,3/6,5_ = 8.8 Hz, H-2, H-6), 7.68 (d, 2H, *J*_2′,3′/6′,5′_ = 7.2 Hz, H-2′, H-6′), 7.64 (d, 1H, *J*_4(3,5)_ = 7.2 Hz, H-4), 7.55 (d, 2H, *J*_3′′,2′′/5′′,6′′ _= 7.6 Hz, H-3′′, H-5′′), 7.51 (t, 2H, *J*_3 (2,4)/5 (4,6)_ = 7.6 Hz, H-3, H-5) 7.48 (d, 2H, *J*_2′′,3′′/6′′,5′′_ = 7.2 Hz, H-2′′, H-6′′), 7.52 (d, 2H, *J*_3′,2′/5′,6′_ = 8.8 Hz, H-3′, H-5′), 5.14 (s, 2H, CH_2_); ^13^C-NMR (100 MHz, DMSO-*d*_6_): *δ*_C_ 194.4 (C=O), 161.8 (C-4′), 137.7 (C-1), 135.5 (C-1′), 132.6 (C-1′′), 132.2 (C-2, C-6), 132.1 (C-2′′, C-6′′), 129.7 (C-3, C-5), 129.6 (C-3′′, C-5′′), 129.2 (C-4), 128.5 (C-4′′), 128.4 (C-2′, C-6′), 114.7 (C-3′, C-5′), 68.7 (CH_2_); EI-MS: *m/z* (rel. abund. %), 324 [M + 2]^+^ (1.9), 322 [M]^+^ (6.1), 125 (100.0), 105 (3.2), 89 (6.7), 78 (8.6); HREI-MS: *m/z* calcd for C_20_H_15_ClO_2_ [M]^+^ 322.0761, found 322.0760.

#### 4.4.10. (4-(4-Methylbenzyloxy)phenyl)(phenyl)methanone (10)

Yield: 86%; m.p. 106–108°C; R*_f_*: 0.49 (ethyl acetate/hexanes, 2:8); IR (KBr, cm^−1^): 3090 (=C–H), 1665 (C=O), 1578 (C=C), 1167 (C–O); ^1^H-NMR (400 MHz, DMSO-*d_6_*): *δ*_H_ 7.75 (d, 2H, *J*_2,3/6,5_ = 8.4 Hz, H-2, H-6), 7.68 (d, 2H, *J*_2′,3′/6′,5′_ = 7.2 Hz, H-2′, H-6′), 7.64 (d, 1H, *J*_4(3,5)_ = 7.2 Hz, H-4), 7.55 (t, 2H, *J*_3 (2,4)/5 (4,6)_ = 7.6 Hz, H-3, H-5), 7.28 (m, 2H, H-3′, H-5′), 7.17 (d, 3H, *J*_4′′(5′′,6′′)/5′′,4′′/6′′,5′′_ = 8.8 Hz, H-4′′, H-5′′, H-6′′), 5.17 (s, 2H, CH_2_), 2.31 (s, 3H, 3′′-CH_3_); EI-MS: *m/z* (rel. abund.%), 302 [M]^+^ (0.9), 141 (3.9), 121 (5.4), 115 (3.2), 105 (100.0), 77 (34.1), 63 (9.2), 51 (4.8); HREI-MS: *m/z* calcd for C_21_H_18_O_2_ [M]^+^ 302.1307, found 302.1305.

#### 4.4.11. (4-(3-Methoxybenzyloxy)phenyl)(phenyl)methanone (11)

Yield: 83%; m.p. 120–122°C; R*_f_*: 0.44 (ethyl acetate/hexanes, 2:8); IR (KBr, cm^−1^): 3150 (=C–H), 1689 (C=O), 1550 (C=C), 1157 (C–O); ^1^H-NMR (300 MHz, DMSO-*d_6_*): *δ*_H_ 7.74 (d, 2H, *J*_2,3/6,5_ = 8.8 Hz, H-2, H-6), 7.68 (d, 2H, *J*_2′,3′/6′,5′_ = 8.4 Hz, H-2′, H-6′), 7.64 (d, 1H, *J*_6′′,5′′_ = 7.2 Hz, H-6′′) 7.55 (t, 2H, *J*_3 (2,4)/5 (4,6)_ = 7.6 Hz, H-3, H-5), 7.33 (t, 1H, *J*_4(3,5)_ = 7.2 Hz, H-4), 7.17 (d, 2H, *J*_3′,2′/5′,6′_ = 8.4 Hz, H-3′, H-5′), 7.03 (m, 2H, H-2′′, H-5′′), 6.91 (dd, 1H, *J*_4′′,5′′ _= 6.4 Hz, *J*_4′′,2′′ _= 1.6 Hz, H-4′′); EI-MS: *m/z* (rel. abund. %), 318 [M]^+^ (74.4), 211 (4.2), 198 (6.4), 169 (2.3), 141 (4.2), 121 (100.0), 105 (16.0), 91 (38.8), 77 (28.1); HREI-MS: *m/z* calcd for C_21_H_18_O_3_ [M]^+^ 318.1256, found 318.1255.

#### 4.4.12. 2-(4-Benzoylphenoxy)-1-(4-bromophenyl)ethanone (12)

Yield: 82%; m.p. 126–128°C; R*_f_*: 0.47 (ethyl acetate/hexanes, 2:8); IR (KBr, cm^−1^): 3390 (=C–H) 1703 (C=O), 1637 (C=O), 1593 (C=C), 1315 (C–O), 560 (=C–Br); ^1^H-NMR (300 MHz, DMSO-*d_6_*): *δ*_H_ 7.79 (d, 2H, *J*_2′′,3′′/6′′,5′′_ = 8.4 Hz, H-2′′, H-6′′), 7.81 (d, 2H, *J*_3′′,2′′/5′′,6′′_ = 8.4 Hz, H-3′′, H-5′′), 7.73 (m, 5H, H-2, H-4, H-6, H-2′, H-6′), 7.56 (t, 2H, *J*_3(2,4)/5(4,6)_ = 7.2 Hz, H-3, H-5), 7.71 (d, 2H, *J*_3′,2′/5′,6′_ = 8.7 Hz, H-3′, H-5′), 5.71 (s, 2H, CH_2_); ^13^C-NMR (100 MHz, DMSO-*d*_6_): *δ*_C_ 194.4 (C=O), 193.2 (C=O), 161.6 (C-4′), 137.6 (C-1), 133.2 (C-1′), 132.1 (C-1′′), 132.0 (C-2, C-6), 131.9 (C-2′′, C-6′′), 129.9 (C-3, C-5), 129.7 (C-3′′, C-5′′), 129.2 (C-4), 128.4 (C-4′′), 128.0 (C-2′, C-6′), 114.6 (C-3′, C-5′), 70.2 (CH_2_); EI-MS: *m/z* (rel. abund.%), 396 [M^+^+2] (3.8), 394 [M]^+^ (3.9), 376 (4.7), 332 (7.7), 239 (7.5), 180 (100.0), 166 (95.3), 155 (3.5), 77 (44.9); HREI-MS: *m/z* calcd for C_21_H_15_BrO_3_ [M]^+^ 394.0205, found 394.0203.

#### 4.4.13. 2-(4-Benzoylphenoxy)-1-(4-nitrophenyl)ethanone (13)

Yield: 84%; m.p. 113–115°C; *R_f:_* 0.49 (Ethyl acetate/hexane, 3 : 7); IR (KBr, cm^−1^): 3393 (=C–H) 1709 (C=O), 1620 (C=O), 1598 (C=C), 1311 (C–O), 760 (N–O); ^1^H-NMR (300 MHz, DMSO-*d_6_*): *δ* 8.40 (d, 2H, *J*_3′′,2′′/5′′,6′′_= 7.5 Hz, H-3′′,H-5′′), 8.27 (d, 2H, *J*_2′′,3′′/6′′,5′′_= 7.5 Hz, H-2′′,H-6′′), 7.74 (d, 2H, *J*_3′,2′/5′,6′ _= 7.5 Hz, H-3′,H-5′), 7.70 (m, 2H, H-3, H-5), 7.64 (m, 1H, H-4), 7.56 (m, 2H, H-2,H-6), 7.17 (d, 2H, *J*_2′,3′/6′,5′_= 7.5 Hz, H-2′, H-6′), 5.80 (s, 2H, CH_2_); EI-MS: *m/z* (rel. abund.%), 361.1 [M]^+^ (41.0), 345 (1.6), 284 (16.6), 211 (17.8), 198 (39.9), 181 (11.2), 150 (100.0), 121 (77.7), 105 (65.9), 77 (42.3); HREI-MS: *m/z* calcd for C_21_H_15_NO_5_ [M]^+^ 361.0950, found 361.0952.

#### 4.4.14. 2-(4-Benzoylphenoxy)-1-(3-methoxyphenyl)ethanone (14)

Yield: 84%; m.p. 153–156°C; R*_f_*: 0.46 (ethyl acetate/hexanes, 2:8); IR (KBr, cm^−1^): 3377 (=C–H), 1697 (C=O), 1635 (C=O), 1598 (C=C), 1313 (C–O); ^1^H-NMR (300 MHz, DMSO-*d_6_*): *δ*_H_ 7.73 (m, 4H, H-2, H-3, H-5, H-6), 7.64 (d, 2H, *J*_2′,3′/6′,5′_ = 8.4 Hz, H-2′, H-6′), 7.56 (m, 4H, H-2′′, H-4′′, H-5′′, H-6′′), 7.28 (d, 1H, *J*_4(3,5)_ = 6.6 Hz, H-4), 7.13 (t, 2H, *J*_3′(2′,4′)/5′(4′,6′)_ = 8.7 Hz, H-3′, H-5′), 5.73 (s, 2H, CH_2_), 3.83 (s, 3H, 3′′-OCH_3_); ^13^C-NMR (100 MHz, DMSO-*d*_6_): *δ*_C_ 194.4 (C=O), 193.8 (C=O), 161.7 (C-4′), 159.5 (C-3′′), 137.7 (C-1), 135.5 (C-1′′), 132.1 (C-1′), 132.0 (C-2, C-6), 130.0 (C-4), 129.7 (C-3, C-5), 129.2 (C-2′, H-6′), 128.4 (C-2′′), 120.3 (C-5′′), 119.8 (C-2′′), 114.4 (C-6′′), 112.3 (C-4′′), 70.3 (CH_2_), 55.4 (3′′-OCH_3_); EI-MS: *m/z* (rel. abund.%), 346 [M]^+^ (18.5), 209 (2.6), 198 (7.8), 150 (2.7), 135 (100.0), 121 (15.8), 107 (19.1), 92 (4.1), 77 (12.0); HREI-MS: *m/z* calcd for C_22_H_18_O_4_ [M]^+^ 346.1205, found 346.1224.

#### 4.4.15. 2-(4-Benzoylphenoxy)-1-(biphenyl-4-yl)ethanone (15)

Yield: 88%; m.p. 100–102°C; R*_f_*: 0.50 (ethyl acetate/hexanes, 2:8); IR (KBr, cm^−1^): 3376 (=C–H), 1692 (C=O), 1636 (C=O), 1589 (C=C), 1310 (C–O); ^1^H-NMR (300 MHz, DMSO-*d_6_*): *δ*_H_ 8.13 (d, 2H, *J*_2′′,3′′/6′′,5′′_ = 8.4 Hz, H-2′′, H-6′′), 7.89 (d, 2H, *J*_2,3/6,5_ = 8.4 Hz, H-2, H-6), 7.78 (m, 7H, H-3, H-4, H-5, H-2′, H-6′, H-3′′, H-5′′), 7.56 (m, 4H, H-3′, H-5′, H-3‴, H-5‴), 7.46 (d, 1H, *J*_4‴(3‴,5‴)_ = 8.7 Hz, H-4‴), 7.15 (d, 2H, *J*_2‴,3‴/6‴,5‴_ = 9.0 Hz, H-2‴, H-6‴), 5.77 (s, 2H, CH_2_); ^13^C-NMR (75.0 MHz, DMSO-*d*_6_): *δ*_C_ 194.4 (C=O), 193.5 (C=O), 161.7 (C-4′), 145.2 (C-1‴), 138.7 (C-1), 137.7 (C-1′), 133.0 (C-4), 132.1 (C-2, C-6), 132.0 (C-2′, C-6′), 129.7 (C-2′′, C-6′′), 129.2 (C-3′′, H-5′′), 129.1 (C-2‴, C-6^‴^), 128.6 (C-3^‴^, C-5^‴^), 128.5 (C-1′), 128.4 (C-4^‴^), 127.3 (C-3, C-5), 127.0 (C-3′, C-5′), 114.6 (C-4′′), 70.3 (CH_2_); EI-MS: *m/z* (rel. abund.%), 392 [M]^+^ (11.6), 315 (0.8), 287 (0.2), 211 (0.2), 181 (100.0), 152 (63.4), 115 (0.9), 105 (39.8), 77 (55.9); HREI-MS: *m/z* calcd for C_27_H_20_O_3_ [M]^+^ 394.1412, found 392.1413.

#### 4.4.16. 2-(4-Benzoylphenoxy)-1-(4-methoxyphenyl)ethanone (16)

Yield: 84%; m.p. 145–147°C; R*_f_*: 0.47 (ethyl acetate/hexanes, 2:8); IR (KBr, cm^−1^): 3372 (=C–H), 1690 (C=O), 1636 (C=O), 1158 (C=C), 1310 (C–O); ^1^H-NMR (300 MHz, DMSO-*d_6_*): *δ*_H_ 8.02 (d, 2H, *J*_2,3/6,5_ = 8.7 Hz, H-2, H-6), 7.73 (m, 4H, H-2′, H-3′, H-5′, H-6′), 7.64 (d, 1H, *J*_4(3,5)_ = 8.7 Hz, H-4), 7.56 (t, 2H, *J*_3′(2′,4′)/5′(4′,6′)_ = 8.7 Hz, H-3′, H-5′), 7.10 (d, 4H, *J*_2′′,3′′/3′′,2′′/6′′,5′′/5′′,6′′)_ = 8.7 Hz, H-2^′′^, H-3^′′^, H-5^′′^, H-6^′′^), 5.77 (s, 2H, CH_2_), 3.85 (4′′-OCH_3_); ^13^C-NMR (75.0 MHz, DMSO-*d*_6_): *δ*_C_ 194.3 (C=O), 192.2 (C=O), 163.6 (C-4′), 161.7 (C-4′′), 137.6 (C-1), 132.0 (C-1′), 131.9 (C-1′′), 130.2 (C-4), 129.6 (C-2, C-6), 129.2 (C-3, C-5), 128.4 (C-2′, H-6′), 127.1 (C-2′′, C-6′′), 114.5 (C-3′, C-5′), 114. (C-3′′, C-5′′), 69.9 (CH_2_), 55.6 (4′′-OCH_3_); EI-MS: *m/z* (rel. abund.%), 346 [M]^+^ (19.9), 198 (5.9), 181 (2.1), 169 (2.1), 152 (3.4), 135 (100.0), 121 (25.0), 107 (18.5), 92 (15.9), 77 (45.7); HREI-MS: *m/z* calcd for C_22_H_18_O_4_ [M]^+^ 346.1205, found 346.1183.

#### 4.4.17. 2-(4-Benzoylphenoxy)-1-(4-chlorophenyl)ethanone (17)

Yield: 83%; m.p. 150–152°C; R*_f_*: 0.45 (ethyl acetate/hexanes, 2:8); IR (KBr, cm^−1^): 3390 (=C–H), 1703 (C=O), 1637 (C=O), 1596 (C=C), 1315 (C–O), 1192 (=C–Cl); ^1^H-NMR (300 MHz, DMSO-*d_6_*): *δ*_H_ 8.05 (d, 2H, *J*_2,3/6,5_ = 8.7 Hz, H-2, H-6), 7.73 (m, 7H, H-4, H-2′, H-3′, H-5′, H-6′, H-3′′, H-5′′), 7.56 (t, 2H, *J*_3(2,4)/5(4,6)_ = 7.8 Hz, H-3, H-5), 7.14 (d, 2H, *J*_2′,3′/6′,5′_ = 8.4 Hz, H-2′, H-6′), 5.72 (s, 2H, CH_2_); ^13^C-NMR (75.0 MHz, DMSO-*d*_6_): *δ*_C_ 194.3 (C=O), 193.1 (C=O), 161.6 (C-4′), 138.7 (C-4′′), 137.6 (C-1), 132.9 (C-1′), 132.1 (C-1′′), 132.0 (C-4), 129.8 (C-2, C-6), 129.7 (C-3, C-5), 129.2 (C-2′, H-6′), 128.9 (C-2′′, C-6′′), 128.4 (C-3′′, C-5′′), 114.6 (C-3′, C-5′), 70.2 (CH_2_); EI-MS: *m/z* (rel. abund.%), 352 [M + 2]^+^ (11.7), 350 [M]^+^ (36.0), 211 (2.7), 198 (4.8), 181 (100.0), 151 (1.2), 105 (19.0), 77 (20.9); HREI-MS: *m/z* calcd for C_21_H_15_ClO_3_ [M]^+^ 350.0710, found 350.0691.

#### 4.4.18. 2-(4-Benzoylphenoxy)-1-(3,4-dichlorophenyl)ethanone (18)

Yield: 81%; m.p. 142–145°C; R*_f_*: 0.45 (ethyl acetate/hexanes, 2:8); IR (KBr, cm^−1^): 3383 (=C–H), 1710 (C=O), 1628 (C=O), 1557 (C=C), 1309 (C–O); ^1^H-NMR (300 MHz, DMSO-*d_6_*): *δ*_H_ 8.26 (d, 1H, *J*_2′′,5′′_ = 1.5 Hz, H-2′′), 7.98 (dd, 1H, *J*_5′′,6′′ _= 6.6 Hz, *J*_5′′,2′′ _= 1.8 Hz, H-5′′), 7.88 (d, 1H, *J*_6′′,5′′_ = 8.4 Hz, H-6′′), 7.73 (d, 2H, *J*_2,3_ = *J*_6,5 _= 8.7 Hz, H-2, H-6), 7.70 (t, 1H, *J*_4(3,5)_ = 8.7 Hz, H-4), 7.64 (d, 2H, *J*_(2′,3′)/(6′,5′)_ = 7.2 Hz, H-2′, H-6′), 7.56 (t, 2H, *J*_3(2,4)/5(4,6)_ = 8.5 Hz, H-3, H-5), 7.16 (d, 2H, *J*_3′,2′/5′,6′_ = 7.3 Hz, H-3′, H-5′), 5.74 (s, 2H, CH_2_); ^13^C-NMR (75.0 MHz, DMSO-*d*_6_): *δ*_C_ 194.4 (C=O), 193.1 (C=O), 161.6 (C-4′), 137.7 (C-3^‴^), 136.0 (C-4^‴^), 133.8 (C-1′), 133.5 (C-1), 132.1 (C-2, C-6), 132.0 (C-2′, C-6′), 130.8 (C-1^‴^), 129.8 (C-2^‴^), 129.3 (H-5′′), 128.4 (C-6^‴^), 127.7 (C-3, C-5), 126.5 (C-3′, C-5′), 114.6 (C-4), 70.3 (CH_2_); EI-MS: *m/z* (rel. abund. %), 388 [M + 4]^+^ (10), 386 [M + 2]^+^ (49), 384 [M]^+^ (76.2), 349 (1.9), 211 (9.2), 198 (19.1), 173 (100.0), 121 (29.9), 105 (33.6), 77 (24.0); HREI-MS: *m/z* calcd for C_21_H_14_Cl_2_O_3_ [M]^+^ 384.0320, found 384.0321.

#### 4.4.19. 2-(4-Benzoylphenoxy)-1-phenylethanone (19)

Yield: 82%; m.p. 150–152°C; R*_f_*: 0.47 (ethyl acetate/hexanes, 2:8); ^1^H-NMR (400 MHz, DMSO-*d_6_*): *δ*_H_ 8.03 (d, 2H, *J*_2,3/6,5_ = 7.2 Hz, H-2, H-6), 7.73 (d, 2H, *J*_2′,3′/6′,5′_ = 9.2 Hz, H-2′, H-6′), 7.70 (m, 4H, H-4, H-2′′, H-4′′, H-6′′), 7.64 (d, 1H, *J*_4(3,5)_ = 7.2 Hz, H-4), 7.59 (m, 4H, H-3, H-5, H-3′′, H-5′′), 7.13 (d, 2H, *J*_3′,2′/5′,6′_ = 8.8 Hz, H-3′, H-5′), 5.74 (s, 2H, CH_2_); EI-MS: *m/z* (rel. abund. %), 316 [M]^+^ (41.7), 239 (1.4), 211 (1.9), 198 (4.9), 121 (5.8), 105 (100.0), 77 (20.4); HREI-MS: *m/z* calcd for C_21_H_16_O_3_ [M]^+^ 316.1099, found 316.1098.

#### 4.4.20. 2-(4-Benzoylphenoxy)-1-*p*-tolylethanone (20)

Yield: 85%; m.p. 220–222°C; R*_f_*: 0.49 (ethyl acetate/hexanes, 2:8); ^1^H-NMR (400 MHz, DMSO-*d_6_*): *δ*_H_ 7.93 (d, 2H, *J*_2,3/6,5_ = 8.4 Hz, H-2, H-6), 7.73 (d, 2H, *J*_2′,3′/6′,5′_ = 8.8 Hz, H-2′, H-6′), 7.69 (d, 2H, *J*_3′,2′/5′,6′_ = 8.0 Hz, H-3′, H-5′), 7.64 (d, 1H, *J*_4(3,5)_ = 7.2 Hz, H-4), 7.55 (t, 2H, *J*_3(2,4)/5(4,6)_ = 7.6 Hz, H-3, H-5), 7.39 (d, 2H, *J*_3′′,2′′/5′′,6′′_ = 8.0 Hz, H-3′′, H-5′′), 7.11 (d, 2H, *J*_2′′,3′′/6′′,5′′_ = 8.8 Hz, H-2′′, H-6′′), 5.73 (s, 2H, CH_2_), 2.39 (s, 3H, CH_3_); EI-MS: *m/z* (rel. abund.%), 330 [M]^+^ (48.0), 253 (1.2), 198 (2.8), 181 (4.1), 152 (10.2), 119 (100.0), 105 (41.2), 91 (60.4), 77 (37.4); HREI-MS: *m/z* calcd for C_22_H_18_O_3_ [M]^+^ 330.1256, found 330.1255.

## Data accessibility

The spectroscopic data are submitted to journal as supporting files. The supplementary data are available at http://dx.doi.org/10.5061/dryad.2r7f832 [31].

## References

[1] Kayaa D, Yalçına FN, Bedir E, Çalışc İ, Steinhauser L, Albert K, Ersöz T 2011 New benzophenone glucosides from the aerial parts of *Gentiana verna* L. subsp. *pontica* (Soltok.) Hayek. Phytochem. Lett. 4, 459–461. (doi:10.1016/j.phytol.2011.08.007)

[2] Trinh BT, Nguyen NTT, Ngo NT, Tran PT, Nguyen LTT, Nguyen LHD 2013 Polyisoprenylated benzophenone and xanthone constituents of the bark of *Garcinia cochinchinensis*. Phytochem. Lett. 6, 224–227. (doi:10.1016/j.phytol.2013.02.004)

[3] Ma XD, Zhang X, Dai HF, Yang SQ, Yang LM, Gu SX, Chen FE 2011 Synthesis and biological activity of naphthyl-substituted (B-ring) benzophenone derivatives as novel non-nucleoside HIV-1 reverse transcriptase inhibitors. Bioorg. Med. Chem. 19, 4601–4607. (doi:10.1016/j.bmc.2011.06.007) 21719299 10.1016/j.bmc.2011.06.007

[4] Pettit GR, Toki B, Herald DL, Verdier-Pinard P, Boyd MR, Hamel E, Pettit RK 1998 Synthesis of phenstatin phosphate. J. Med. Chem. 41, 1688–1695. (doi:10.1021/jm970644q) 9572894 10.1021/jm970644q

[5] Khanum SA, Begum BA, Girish V, Khanum NF 2010 Synthesis and evaluation of benzophenone-n-ethyl morpholine ethers as anti-inflammatory agents. Int. J. Biomed. Sci. 6, 60–65. 23675177 PMC3614727

[6] Sakunpak A, Panichayupakaranant P 2012 Antibacterial activity of Thai edible plants against gastrointestinal pathogenic bacteria and isolation of a new broad spectrum antibacterial polyisoprenylated benzophenone chamuangone. Food Chem. 130, 826–831. (doi:10.1016/j.foodchem.2011.07.088)

[7] Yamazaki Y, Sumikura M, Masuda Y, Hayashi Y, Yasui H, Kiso Y, Neuteboom S 2012 Synthesis and structure–activity relationships of benzophenone-bearing diketopiperazine-type anti-microtubule agents. Bioorg. Med. Chem. 20, 4279–4289. (doi:10.1016/j.bmc.2012.05.059) 22727370 10.1016/j.bmc.2012.05.059

[8] Schmitt MR, Carzaniga R, Cotter HVT, O'Connell R, Hollomon D 2006 Microscopy reveals disease control through novel effects on fungal development: a case study with an early-generation benzophenone fungicide. Pest Manag. Sci. 62, 383–392. (doi:10.1002/ps.1177) 16602068 10.1002/ps.1177

[9] Arshia A, Khan A, Khan KM, Saad SM, Siddiqui NI, Javaid S, Choudhary MI 2016 Synthesis and urease inhibitory activities of benzophenone semicarbazones/thiosemicarbazones. Med. Chem. Res. 25, 2666–2679. (doi:10.1007/s00044-016-1673-0)

[10] Jantan I, Saputri FC 2012 Benzophenones and xanthones from *Garcinia cantleyana* var. cantleyana and their inhibitory activities on human low-density lipoprotein oxidation and platelet aggregation. Phytochemistry 80, 58–63. (doi:10.1016/j.phytochem.2012.05.003) 22640928 10.1016/j.phytochem.2012.05.003

[11] Shi JB, Chen LZ, Wang Y, Xiou C, Tang WJ, Zhou HP, Liu XH, Yao QZ 2016 Benzophenone-nucleoside derivatives as telomerase inhibitors: design, synthesis and anticancer evaluation *in vitro* and *in vivo*. Eur. J. Med. Chem. 124, 729–739. (doi:10.1016/j.ejmech.2016.09.011) 27639364 10.1016/j.ejmech.2016.09.011

[12] Zabiulla, Neralagundi HGS, Begum AB, Prabhakar BT, Khanum SA 2016 Design and synthesis of diamide-coupled benzophenones as potential anticancer agents. Eur. J. Med. Chem. 115, 342–351. (doi:10.1016/j.ejmech.2016.03.040) 27027818 10.1016/j.ejmech.2016.03.040

[13] Youn UJ, Sripisut T, Miklossy G, Turkson J, Laphookhieo S, Chang LC 2017 Bioactive polyprenylated benzophenone derivatives from the fruits extracts of *Garcinia xanthochymus*. Bioorg. Med. Chem. Lett. 27, 3760–3765. (doi:10.1016/j.bmcl.2017.06.073) 28729053 10.1016/j.bmcl.2017.06.073

[14] Shiraishi Y, Furubayashi Y, Nishimura G, Hirai T 2007 Sensitized luminescence of Eu and Tb macrocyclic complexes bearing benzophenone antennae. J. Lumin. 126, 68–76. (doi:10.1016/j.jlumin.2006.05.007)

[15] Shiraishi Y, Furubayashi Y, Nishimura G, Hirai T 2007 Sensitized luminescence properties of dinuclear lanthanide macrocyclic complexes bearing a benzophenone antenna. J. Lumin. 127, 623–632. (doi:10.1016/j.jlumin.2007.03.021)

[16] Wang K, Yang K, Yu Q 2014 Novel polymeric photoinitiators with side-chain benzophenone: facile synthesis and photopolymerization properties without coinitiator. Prog. Org. Coat. 77, 1929–1934. (doi:10.1016/j.porgcoat.2014.06.026)

[17] Karahan Ö, Balta DK, Arsu N, Avci D 2014 Synthesis and evaluations of novel photoinitiators with side-chain benzophenone, derived from alkyl *α*-hydroxymethacrylates. J. Photochem. Photobiol. A: Chem. 274, 43–49. (doi:10.1016/j.jphotochem.2013.09.010)

[18] Den Boer M, Argaw D, Jannin J, Alvar J 2011 Leishmaniasis impact and treatment access. Clin. Microbiol. Infect. 17, 1471–1477. (doi:10.1111/j.1469-0691.2011.03635.x) 21933305 10.1111/j.1469-0691.2011.03635.x

[19] World Health Organization, Online: http://www.who. int emc diseases leish leisdis. html (accessed, 2003 14).

[20] David CV, Craft N 2009 Cutaneous and mucocutaneous leishmaniasis. Dermatol. Ther. 22, 491–502. (doi:10.1111/j.1529-8019.2009.01272.x) 19889134 10.1111/j.1529-8019.2009.01272.x

[21] Reithinger R, Dujardin JC, Louzir H, Pirmez C, Alexander B, Brooker S 2007 Cutaneous leishmaniasis. Lancet Infect. Dis. 7, 581–596. (doi:10.1016/S1473-3099(07)70209-8) 17714672 10.1016/S1473-3099(07)70209-8

[22] Costa CHN, Peters NC, Maruyama SR, de Brito EC Jr, de Miranda Santos IKF 2011 Vaccines for the leishmaniases: proposals for a research agenda. Trop. Dis. 5, e943 (doi:10.1371/journal.pntd.0000943) 10.1371/journal.pntd.0000943 PMC3066138 21468307

[23] Croft SL, Sundar S, Fairlamb AH 2006 Drug resistance in leishmaniasis. Clin. Microbiol. Rev. 19, 111–126. (doi:10.1128/CMR.19.1.111-126.2006) 16418526 10.1128/CMR.19.1.111-126.2006 PMC1360270

[24] Maciel-Rezende CM, de Almeida L, Costa ÉDM, Pires FR, Alves KF, Junior CV, dos Santos MH 2013 Synthesis and biological evaluation against *Leishmania amazonensis* of a series of alkyl-substituted benzophenones. Bioorg. Med. Chem. 21, 3114–3119. (doi:10.1016/j.bmc.2013.03.045) 23623672 10.1016/j.bmc.2013.03.045

[25] Taha M, Baharudin MS, Ismail NH, Khan KM, Jaafar FM, Siddiqui S, Choudhary MI 2013 Synthesis of 2-methoxybenzoylhydrazone and evaluation of their antileishmanial activity. Bioorg. Med. Chem. Lett. 23, 3463–3466. (doi:10.1016/j.bmcl.2013.03.051) 23608761 10.1016/j.bmcl.2013.03.051

[26] Khan KM, Taha M, Naz F, Khan M, Rahim F, Perveen S, Choudhary MI 2011 Synthesis and *in vitro* leishmanicidal activity of disulfide derivatives. Med. Chem. 7, 704–710. (doi:10.2174/157340611797928460) 22313310 10.2174/157340611797928460

[27] Khan KM, Mughal UR, Ambreen N, Samreen, Perveen S, Choudhary MI 2010 Synthesis and leishmanicidal activity of 2,3,4-substituted-5-imidazolones. J. Enz. Inhib. Med. Chem. 25, 29–37. (doi:10.3109/14756360902932768) 10.3109/14756360902932768 20030507

[28] Meshram HM, Goud PR, Reddy BC, Kumar DA 2010 Triton B-mediated efficient and convenient alkoxylation of activated aryl and heteroaryl halides. Synth. Commun. 40, 2122–2129. (doi:10.1080/00397910903219518)

[29] Pathak A, Rajput CS, Bora PS, Sharma S 2013 DMC mediated one-pot synthesis of biaryl ketones from aryl carboxylic and boronic acids. Tetrahedron Lett. 54, 2149–2150. (doi:10.1016/j.tetlet.2013.02.038)

[30] Atta-ur-Rahman, Choudhary MI, Thomsen WJ 2001 Bioassay techniques for drug development. Amsterdam, The Netherlands: Harwood Academic.

[31] Arshia, Ahad F, Ghouri N, Kanwal, Khan K, Perveen S, Choudhary M 2018 Data from: Synthesis of 4-substituted ethers of benzophenone and their antileishmanial activities Dryad Digital Repository. (doi:10.5061/dryad.2r7f832) 10.1098/rsos.171771 PMC5990808 29892370

